# *In Silico* Screening of Aptamers Configuration against Hepatitis B Surface Antigen

**DOI:** 10.1155/2019/6912914

**Published:** 2019-06-26

**Authors:** Mohamad Zulkeflee Sabri, Azzmer Azzar Abdul Hamid, Sharifah Mariam Sayed Hitam, Mohd. Zulkhairi Abdul Rahim

**Affiliations:** ^1^Bioengineering Section, Universiti Kuala Lumpur Malaysian Institute of Chemical and Bioengineering Technology (UniKL MICET), Lot 1988, Bandar Vendor Taboh Naning, 78000 Alor Gajah, Melaka, Malaysia; ^2^Department of Biotechnology, Kulliyyah of Science, International Islamic University Malaysia (IIUM), Bandar Indera Mahkota, 25200 Kuantan, Pahang, Malaysia; ^3^Technical Foundation Section, Universiti Kuala Lumpur Malaysian Institute of Chemical and Bioengineering Technology (UniKL MICET), Lot 1988, Bandar Vendor Taboh Naning, 78000 Alor Gajah, Melaka, Malaysia

## Abstract

Aptamer has been long studied as a substitute of antibodies for many purposes. However, due to the exceeded length of the aptamers obtained* in vitro*, difficulties arise in its manipulation during its molecular conjugation on the matrix surfaces. Current study focuses on computational improvement for aptamers screening of hepatitis B surface antigen (HBsAg) through optimization of the length sequences obtained from SELEX. Three original aptamers with affinity against HBsAg were truncated into five short hairpin structured aptamers and their affinity against HBsAg was thoroughly studied by molecular docking, molecular dynamics (MD) simulation, and Molecular Mechanics Poisson-Boltzmann Surface Area (MMPBSA) method. The result shows that truncated aptamers binding on HBsAg “a” determinant region are stabilized by the dynamic H-bond formation between the active binding residues and nucleotides. Amino acids residues with the highest hydrogen bonds hydrogen bond interactions with all five aptamers were determined as the active binding residues and further characterized. The computational prediction of complexes binding will include validations through experimental assays in future studies. Current study will improve the current* in vitro* aptamers by minimizing the aptamer length for its easy manipulation.

## 1. Introduction

Hepatitis B is a potentially life-threatening disease which majorly affects the liver, caused by the hepatitis B virus. The disease has been acknowledged as a major health problem which can cause a high risk of death from cirrhosis and liver cancer. It is estimated that 240 million people are chronically infected with hepatitis B, and almost 686 000 people died yearly due to the complications caused by the infection [1].

Morphologically, hepatitis B virion consists of outer lipid envelope and an icosahedral nucleocapsid core composed of protein (HBcAg) [2]. The nucleocapsid encloses the viral DNA and a DNA polymerase that has reverse transcriptase activity similar to retroviruses. The outer envelope contains embedded proteins which are involved in viral binding of and entry into the susceptible cells (HBsAg) [3]. Surface and core proteins of hepatitis B virus consist of molecular recognition patterns that can be detected by the antigen presenting cells such as helper T cells and trigger the production of the antibodies through B cells. HBsAg is highly recognized by the antigen presenting cells and exists in several subtypes identified by its three determinants: a, d, and y [4]. The “a” determinant is the most crucial part of HBsAg as it is the immune dominant and immune protective determinant and therefore functions as the target for diagnosis and vaccination [5]. The “a” determinant is located within the major hydrophilic region (MHR) of HBsAg, specifically of amino acids residues 99-169 out of 226-residues. At least three anti-HBsAg monoclonal antibodies which recognized the epitopes had been known with mixed results in their sensitiveness of detecting the wild-type and mutants HBsAg.

Despite the availability of anti-HBsAg monoclonal antibody (MAb) in the laboratories, production of hybridomas in having MAb requires tedious procedures involving* in vivo* applications. High contamination risk through dealing with animals, risk of animal dying prematurely, and failure to clone the hybridomas which requires special facilities and media for animal cell culture made the development of hepatitis B detection though ELISA and antibody-antigen related procedure are costly in the less developed region of the world. In addition to the economical disadvantages, the chemical structure of antibodies which consists of proteins, without having proper chemical modifications, is normally subjected to the heat-labile properties and is sensitive to the macro- and microenvironment. Slight changes in temperature, pH, and ionic distribution in the blood might contribute to the dysfunctional of the antibodies. Therefore, the availability of the aptamers which consists of more stable and robust nucleic acids provides a platform of robust detectors which are highly precise and durable to the changes of the environment in comparison with their antibodies counterpart. While screening aptamers through conventional methods such as SELEX and NECEEM are timely consuming and require high-end laboratory setup and access to large DNA library database, screening aptamers through computational docking and molecular dynamics study are highly sorted. One of the approaches to incorporate computational study in designing aptamers includes introducing Random Filtering methods to selectively increase the number of five-way junctions in RNA/DNA pools, thus creating a potentially high nucleotides stem-loop number systematic library for SELEX application [6]. Another study involved the selection for RNA structure based on the thermodynamically stable secondary structure formation and performed the nucleic acids docking on selected ligands through high-throughput virtual screening procedures [7]. Therefore, as the computing power had increased tremendously in the current decade, the application of computational simulation for aptamer structure prediction and docking screening had become crucial.

The sequence of aptamers against hepatitis B surface antigen (HBsAg) had been identified earlier in another study [8]. However, the length of these aptamers (80-90 mer) will interfere with its manipulation, such as in conjugation on the nanoparticles matrices. If the length of aptamers is too long, it will cause the steric hindrance effect in the aptamer-nanoparticles conjugation process, thus causing ineffective conjugation and decreasing product yield [9]. Long aptamers also tend to encapsulate the surface of the nanostructures in the process, thus limiting the number of molecules able to bind on the nanoparticles surface. In the targeted organ for diagnostics and therapeutics application, long oligonucleotides are associated with the rapid clearance of oligonucleotides-conjugated nanoparticles in the liver and spleen while increasing the nonspecific interactions with positively charged nanoparticles. This cause decreases sensitivity of long aptamers. Current study provides a platform for the postselection process after SELEX, where the aptamer region with high affinity to the antigen is stabilized based on computational analysis tools such as molecular docking and MD simulations, enabling the unnecessary region to be truncated out and keeping the aptamers length short to retain its function.

## 2. Materials and Methods

### 2.1. Structure Modelling of HBsAg

The initial atomic coordinates of hepatitis B antigen subtype* adw* were designed based on the amino acids sequence retrieved from the UniProt KB database (Uni Prot ID: P03141), due to the unavailability of the crystallized structure in RCSB Protein Data Bank. The structure of HBs antigen was predicted by I-TASSER program (https://zhanglab.ccmb.med.umich.edu/I-TASSER/) [10] and the structure model was visualized using PyMOL (version 1.7.0.0-1, Schrödinger, LLC). The missing side-chain atoms were reconstructed using DeepView/Swiss-PdbViewer (https://spdbv.vital-it.ch/) [11]. The tertiary structure of the antigen was then submitted to PROCHECK web server (http://servicesn.mbi.ucla.edu/PROCHECK/) [12] which generates the Ramachandran plot of protein backbone and to ProSA-Web (https://prosa.services.came.sbg.ac.at/prosa.php) where its range of application includes error recognition in experimentally determined structures, theoretical models, and protein engineering by using the Z-score function [13]. The structure optimization of HBsAg model was performed by equilibrating the structure in SPC/E water box [14] using GROMACS (version. 5.1.1, University of Groningen) for 20 ns, under CHARMM27 force field [15] for global energy minimization and position restrain for 100 ps. The final step of validation was the packing quality of each residue as evaluated in the Verify3D server, which represents the profile achieved with respect to the residues [16, 17]. The validated HBsAg structure was finally employed for molecular docking and molecular dynamics studies.

### 2.2. Structural Modelling of Anti-HBsAg Aptamers Hairpin Region

The sequences for three aptamers against HBsAg were obtained from the previous study [8]. The aptamers (H01, H02, and H03) two-dimensional structures were first designed using Mfold (http://unafold.rna.albany.edu/?q=mfold) to determine the loop and hairpin structures [18]. Next, five shorter aptamers with two aptamers from each H01 and H02 and one aptamer from H03 with the length between 9 and 10 nucleotides were designed based on the loop-hairpin structures combination available inside the H01, H02, and H03 full structures (Table 1). Two-dimensional structure of aptamer in Vienna format obtained from Mfold was then submitted to the RNAComposer (http://rnacomposer.cs.put.poznan.pl/) to obtain the three-dimensional structures [19]. The hydroxyl in the deoxyribose and methyl functional group in thymine inside the aptamers structure were added manually using PyMOL “add molecules” and “add group” functions, before energy minimized for 100 ps in GROMACS. The root-mean-squares of each aptamer three-dimensional structure before and after the docking were analyzed using “RMS” function in PyMOL. Even though the three-dimensional structure of single strand DNA was designed based on the RNA structures from the knowledge-based RNA conformation in the RNAComposer, a study to proof the reliability of the designed DNA had been performed where 25 single strand DNA where nucleotide length varies from 7 to 27 nucleotides were designed using the RNAComposer and edited following the aforementioned procedure and compared with actual crystallized DNA structure deposited in the RCSB Databank. Minimal differences of RMSD (3.0Å-10Å) were observed between the DNA designed by above pipeline and its crystallized structures (data not shown).

### 2.3. Aptamer-HBsAg Molecular Docking

Initially, the designed HBsAg structure was minimized and equilibrated using MD for 20 ns. For docking, polar hydrogen atoms were added to HBsAg and its nonpolar hydrogen atoms were merged using AutoDock Tools (http://mgltools.scripps.edu) [20]. The grid box with a dimension of 40 × 40 × 40 points was set around the “a” determinant region (amino acids 99-169) to wholly cover the protein epitope for the docking. For the aptamers, all bonds were set as rotatable. The molecular docking between each single aptamer and HBsAg was conducted using AutoDock Vina, which combines certain advantages of knowledge-based potentials and empirical scoring functions: it extracts empirical information from both the conformational preferences of the receptor-ligand complexes and the experimental affinity measurements [21]. Each aptamer-HBsAg complex with the lowest docked energy was selected as the best conformation. Molecular interactions between HBsAg and each aptamer complexes conformation such as the hydrogen bonds and the bond lengths were analyzed using PyMOL (ver. 1.7.0.0-1, Shrödinger LLC)), Discovery Studio Visualizer (ver. 16.1.0.15350, BIOVIA), and VMD (ver. 1.9.3, University of Illinois).

### 2.4. Molecular Dynamics (MD) Simulation of Complexes

Detailed molecular dynamics simulations using the complex structures were conducted with the CHARMM27 using GROMACS program [22]. CHARMM27 force field has been shown to be effective in protein modelling and used in several molecular dynamics simulations of DNA [23]. Similarly as HBsAg structure optimization MD step, each complex were solvated in a cubic box and keeping a distance of 1.2 nm between the complex and the edge of the solvated box. Sodium and chloride ions were added to neutralize the charge of the system and then were energy minimized using the steepest descent algorithm. In all simulations the condition was set at the room temperature (300 K) and the atmospheric pressure (1 bar) to closely mimic the general experiment conditions. The NVT thermal equilibration was done with a constrained structure and a velocity-rescale thermostat specific to GROMACS. Subsequently, NPT pressure equilibration was applied with the same velocity-rescale temperature coupling in addition to the Parrinello−Rahman pressure coupling. The fully temperature and pressure equilibrated system was then used as the initial configuration for the MD production dynamic analysis. All simulations were conducted using a 2 fs time step. In order to verify the robustness of the results multiple simulations of the HBsAg-aptamer complexes combinations were conducted for a minimum of 20 ns following the same MD procedure. The results were then analyzed using common GROMACS functions such as RMSD and RMSF, while the formation of hydrogen bonds between each aptamer and HBsAg was analyzed using GROMACS “gmx_hbond” functions. Each complex total energy was calculated using “gmx_energy” function, while the distance between aptamers and HBsAg was measured using the “gmx_pairdist” function. The binding free energy between the HBsAg and each aptamer was calculated using the “g_mmpbsa” GROMACS function which implements the MMPBSA method [24]. A total of 250 snapshots were extracted from the trajectories between 15 and 20 ns simulation time and used for MMPBSA binding energy calculations. Hydrogen bond occupancy for each complex was calculated using H-bond occupancy function in VMD.

## 3. Results and Discussions

### 3.1. HBsAg* In Silico* Modelling

In current study I-TASSER server was employed to elucidate the HBsAg structure, since the crystallized structure of HBsAg is not available in the PDB due to its experimental difficulties. I-TASSER had predicted the formation of four helical structures in the HBs antigen. The helices domain are in amino acids 9-39 (I), 72-100 (II), 155-184 (III), and 188-223 (IV). Based on study by Gazina [25] and another independent study by Glebe and Bremer [26], S protein of HBs antigen consists of four transmembrane helical domains (Table 2).

**Figure 1:**
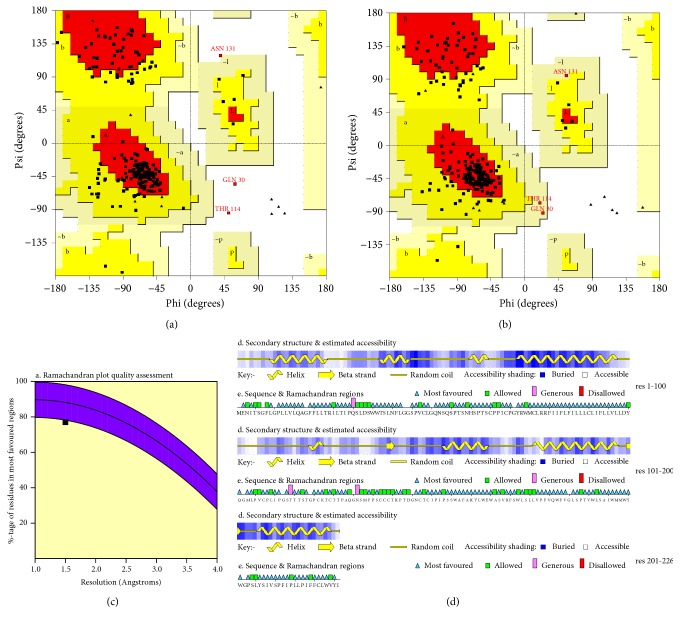
HBs antigen Ramachandran plot generated using PROCHECK web server before (a) and after 20 ns MD (b). A, B, and L: most favoured regions; a, b, l, and p: additional allowed regions; ~a, ~b, ~l, and ~p: generously allowed regions; white areas are the disallowed regions. Glycine residues are shown in triangle. (c) represents percentage of residues that are most favoured in the Ramachandran plot of minimized HBsAg structure which is 77% at 1.5Å resolution. (d) shows the secondary structure formation, where the “a” determinant region (a.a 99-169) majorly consists of random coil structure. After 20 ns MD, out of 226 amino acids there are no amino acids located in the disallowed region while only Q30, T114, and N131 amino acids are in the generously allowed region.

The main Ramachandran plots for HBs antigen structure before and after the MD optimization are shown. The three amino acids in the disallowed region, GLN30, THR114, and ASN131, required optimization of their folding in the structure. After 20 ns MD of HBsAg, all the residues are in the allowed region although minimal increase from 76.5% to 77.0% is observed in the most favoured region (Figure 1). The HBsAg optimized structure after MD has 77% residues in the most favoured region, 21.4% residues in allowed region, and 1.6% residues in generously allowed region while no residues located in the disallowed region. The maximum deviation of the residues was 5.4Å with zero bad contacts.

**Figure 2:**
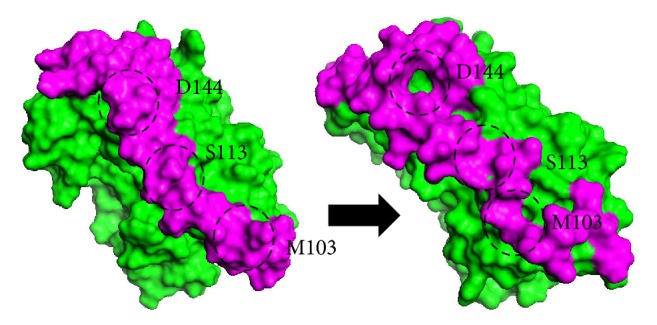
Three-dimensional structure of HBsAg before (left) and after 20 ns MD simulation (right). Magenta colour shows the epitope region of HBsAg which undergoes an obvious conformational change after the whole course of simulation. The formation of anchor-like binding sites along the epitope suggests the location of aptamer binding.

The visual inspection on HBsAg structures in Figure 2 shows the conformational change of HBsAg which happened after 20 ns is highly visible. After 20 ns, the cavity was seen to form near the cysteine rich region of C121, C124, T125, C136-C138, and D144 residues. This deformation suggesting the location for aptamer to bind appeared somewhere else in the epitope, due to the changes of the Connolly surface as the cysteine rich region of HBsAg might repel the negatively charged nucleotides as both the cysteine residues and the aptamer carry the similar negative charges [27]. The conformation of HBsAg near S113 and M103 (Figure 2, in circle) formed an anchor-like inward curve region which enables the aptamers to bind tightly compared to the smooth Connolly surface conformation before the MD simulation [28].

**Figure 3:**
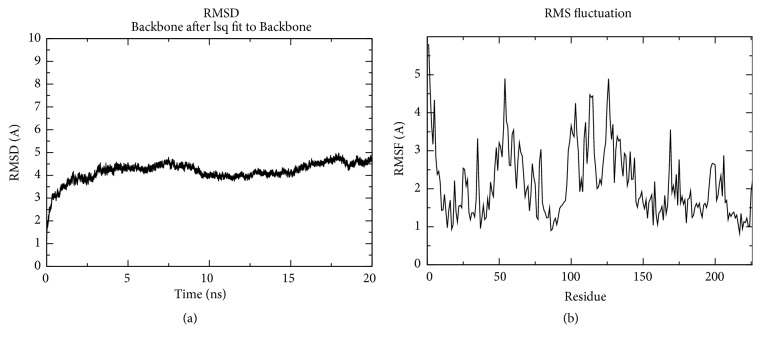
Graphical representation of HBsAg RMSD backbone for 20 ns (a) and fluctuations of HBsAg backbone (RMSF) for 20 ns (b). HBsAg was stable after 3-4 ns simulation, and the highly movable residues were locating near 50 a.a and in “a” determinant region (99-150 a.a).

Figure 3(b) shows that the residue at N-terminal more fluctuates compared to the residue at the C-terminal regions, explainable through the RMSF values. It was discovered that during the dynamics simulations few fluctuations gone beyond 2 Å except for the “a” determinant region amino acids (99-150) and amino acids 44-69. Total proteins show that even less fluctuations exceeded 2.0 Å for the whole 20 ns simulation, as shown in Figure 3. The residues 44–69 with fluctuations close to 3.0-4.0 Å observed in the dynamics plots were located close to the “a” determinant region, which consists of loops structure. Residues Cys107 and Cys121 are the most stable amino acids, showing less than 1 Å fluctuations. Stability of other regions in the HBsAg overall structure is based on the formation of transmembrane helices, thus enforcing the protein to stay in the closed compacted globular structure as observed in the protein's radius of gyration (data not shown).

**Figure 4:**
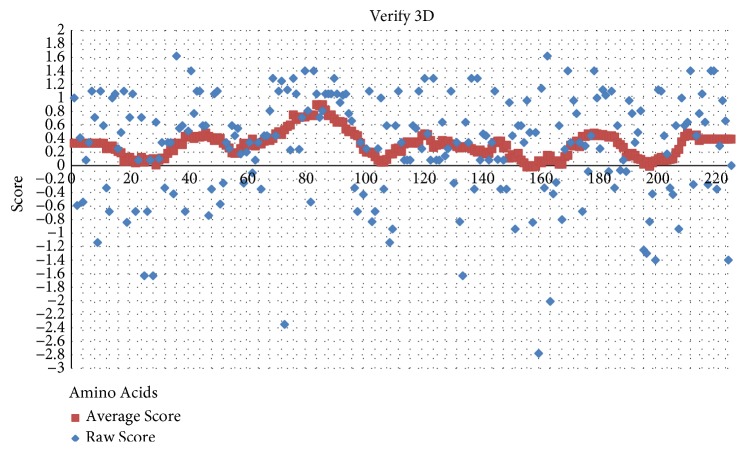
The 3D profiles of HBs antigen model that was generated using Verify3D server. Overall compatibility score above zero for the average score (blue-grey) indicates residues are reasonably folded. Most of the residues are located above score 0.2.

The compatibility score above zero in the Verify3D graph is corresponding to the acceptable side-chain environments (Figure 4). The averaged score for all residues was in the positive value, with 80% of residues being over 0.2. This suggests that the model has overall self-consistency in terms of sequence-structure compatibility.

### 3.2. Three-Dimensional Conformation and Docking of Anti-HBsAg Aptamers Hairpin Domain

**Figure 5:**
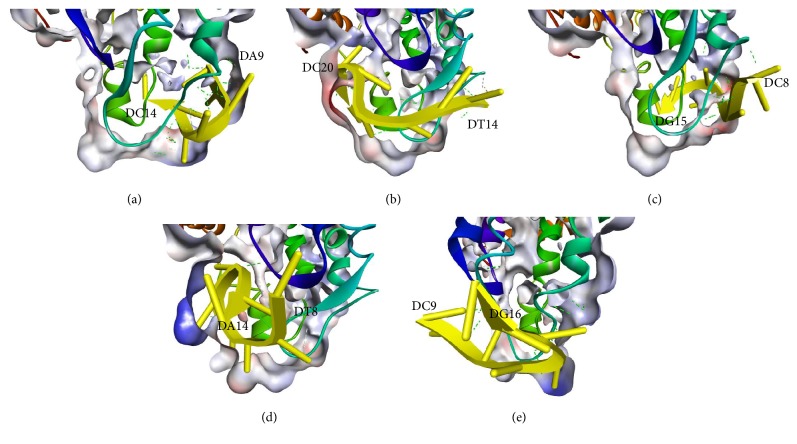
Illustrations of the representative aptamers poses (a) H01a, (b) H01b, (c) H02a, (d) H02b, and (e) H03a (all in yellow) obtained after docking to HBsAg. One part of “a” determinant region is shown in turquoise, where most of the aptamer docking occurred. Intermolecular polar contacts are shown as dashed lines.

Figure 5 shows the docking pose of each aptamer against the “a” determinant region of HBsAg. Docking poses and conformations of each aptamer on the structure show that the lowest-energy conformer predicted by Vina for each ligand was not necessarily the best pose given the lowest RMSD. The whole aptamer docked pose RMS value against the initial truncated hairpin structure is provided in Table 3. It shows that the RMS value of docked poses varies between 6.0 and 8.0 Å. When all the bonds in the aptamer backbone were set to be rotatable, each atom will be able to rotate freely to interact with the receptor (HBsAg) surface following the force-field docking algorithm in Vina, thus also causing the break of the Watson-Crick base pairing at the edge of the hairpin structure. Although the RMS value shows high flexibility of the hairpin aptamer towards the protein receptor, the number of Watson-Crick base pairs in the actual aptamers conformation is still high, which is around 5-6 pairs (from aptamers sequence in Table 1). This enables the hairpin structure to be well maintained during the actual docking. Therefore, although the aptamers active conformer or that closest to the bioactive form was in many cases assumed to be in the hairpin structure [29], it is not every time the conformation of the aptamer in the docked structure may be in the active conformation as in Table 3 of Vina score where each complex Vina score was only slightly different. H03a tends to stay in the hairpin conformation compared to other aptamers. This is visible by the low RMS value in docking (Table 3) and significantly high Vina score (-22.175 kJ/mol). In other words, aptamers with high structure flexibility such as H01b (RMS value 8.944) were shown to give better Vina binding energy (-31.798 kJ/mol).

The conformational analysis of docked structures shows that all aptamers bind to the specific locus in the HBsAg epitope and formed hydrogen bonds with the specific aptamer binder residues in addition to the intramolecular hydrophobic interactions. At least five active binding residues which function as the hydrogen donors towards aptamer were observed from almost all of the aptamer docking, namely, Lys122, Ala128, Gly130, Ser132, Gln129, and Asn132. These residues maintained the aptamers position in docking, which is observable through MD simulation. Further characterization of these aptamer binder residues for the whole course of 20 ns simulation is also discussed in the MD section.

### 3.3. MD Simulation of Aptamer-Protein Complexes

#### 3.3.1. RMSD and Intermolecular Distance

**Figure 6:**
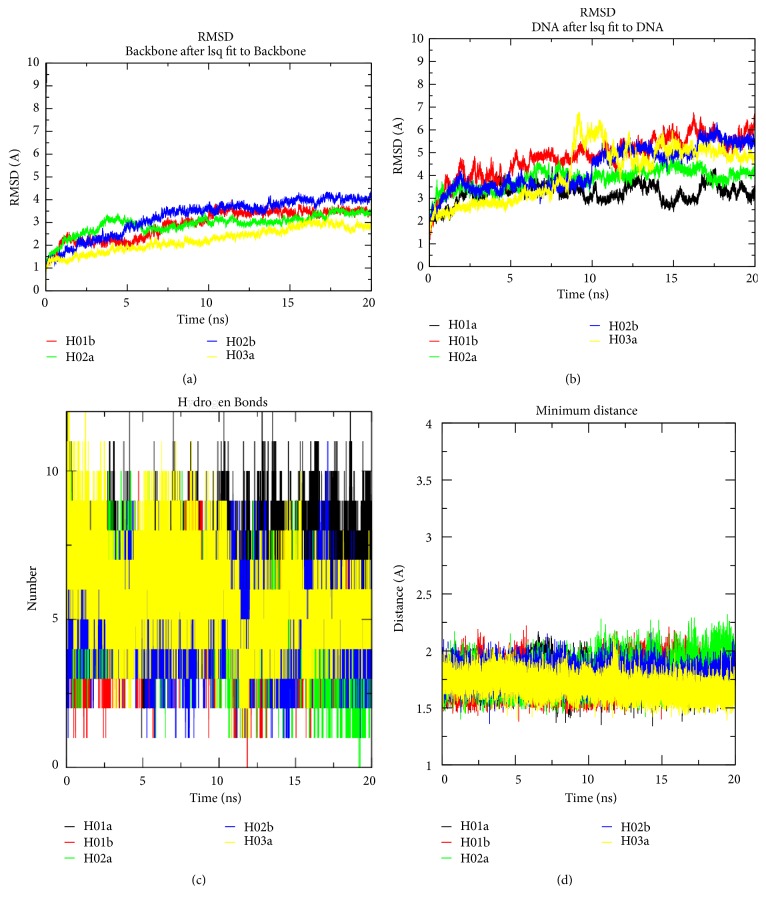
Molecular dynamics trajectories of (a) RMSD of HBsAg in complexes, (b) RMSD of aptamers in complexes, (c) number of intermolecular hydrogen bonds, and (d) minimum distance between HBsAg and aptamers for the 20 ns duration. Each aptamer-HBsAg complex reached equilibrium after 7-8 ns of simulation except H03a, which is after 12 ns of MD. The aptamers vibrate heavily even after binding to HBsAg (b); however the intermolecular H-bond formed between aptamer and HBsAg increases the complex stability (c) and maintains their distance to be in between 1.5 and 2.0 Å (d).

Root-mean-square-deviation (RMSD) calculation of docked HBsAg and each aptamer separately shows that HBsAg is more stable for the whole course of 20 ns simulation, although the conformation starts to reach equilibrium after 7 ns of simulation (Figure 6(a)). The observations were also similarly found in other studies of protein-DNA complex [30] which showed that protein-DNA complex binding equilibrates at 12-20 ns, thus justifying 20 ns as the current threshold of the protein-DNA complex simulation. The RMSD values of the docked HBsAg were highly stable, as they only fluctuate less than 2 Å at each docking complex after 20 ns MD simulation (data not shown). In comparison, aptamers have less after-docked stability, where the docked aptamers RMSD deviate in between 2 and 4 Å of the initial aptamers conformation. It was also noticeable that only H03a had taken more time to reach its complex stability which fluctuates up to 4 Å and only reached equilibrium after 12 ns of MD (Figure 6(b)).

Due to hairpin conformation of H03a, the surface interaction between aptamer and HBsAg remains limited compared to its other counterparts. This also shows that although hairpin structure is always considered as the optimum conformation to stabilize the aptamer itself, it is not necessarily the optimum conformation for binding. Experimental procedure such as that performed in another study [31] is still required to understand the DNA hairpin effects on aptamer-protein binding. The overall number of hydrogen bonds formed between H03a and HBsAg is also shown to decrease over the 20 ns period, in comparison with other aptamer-HBsAg complexes. Nucleotide in aptamers has the ability to form salt bridges or hydrogen bonds with the receptor; in this case HBsAg is more crucial to maintain the dynamical interactions between the two molecules.

The number of dynamic hydrogen bonds formed between HBsAg and each aptamer for 20 ns although it differs shares a mean between 5 and 6 hydrogen bonds as in Figure 6(c). Although it is not excessively high, it shows that the number is sufficient to give a constant minimum distance value over the whole MD period and to maintain the overall distance between the aptamer and HBsAg at an average of 1.75 Å. The active binding residues in HBsAg play an important role in maintaining the number of hydrogen bonds within the 20 ns simulation.

#### 3.3.2. Intermolecular Hydrogen Bond Occupancy by the Active Binding Residues

Active binding residues are the amino acids residues which maintained the intermolecular hydrogen bonds formed between aptamers and protein in the docking poses and throughout the MD simulation [27]. In other words, although the aptamers were dynamically moving during the binding, these bonds will keep holding the aptamer in-place, thus affecting its affinity. The intermolecular hydrogen bonds occupancy is considered a benchmark in determining the active binding residues. By understanding the hydrogen bond occupancy of aptamer-HBsAg complex during the molecular simulation time frame, the important interactions between HBsAg active binding residues and the aptamer can be observed.

**Figure 7:**
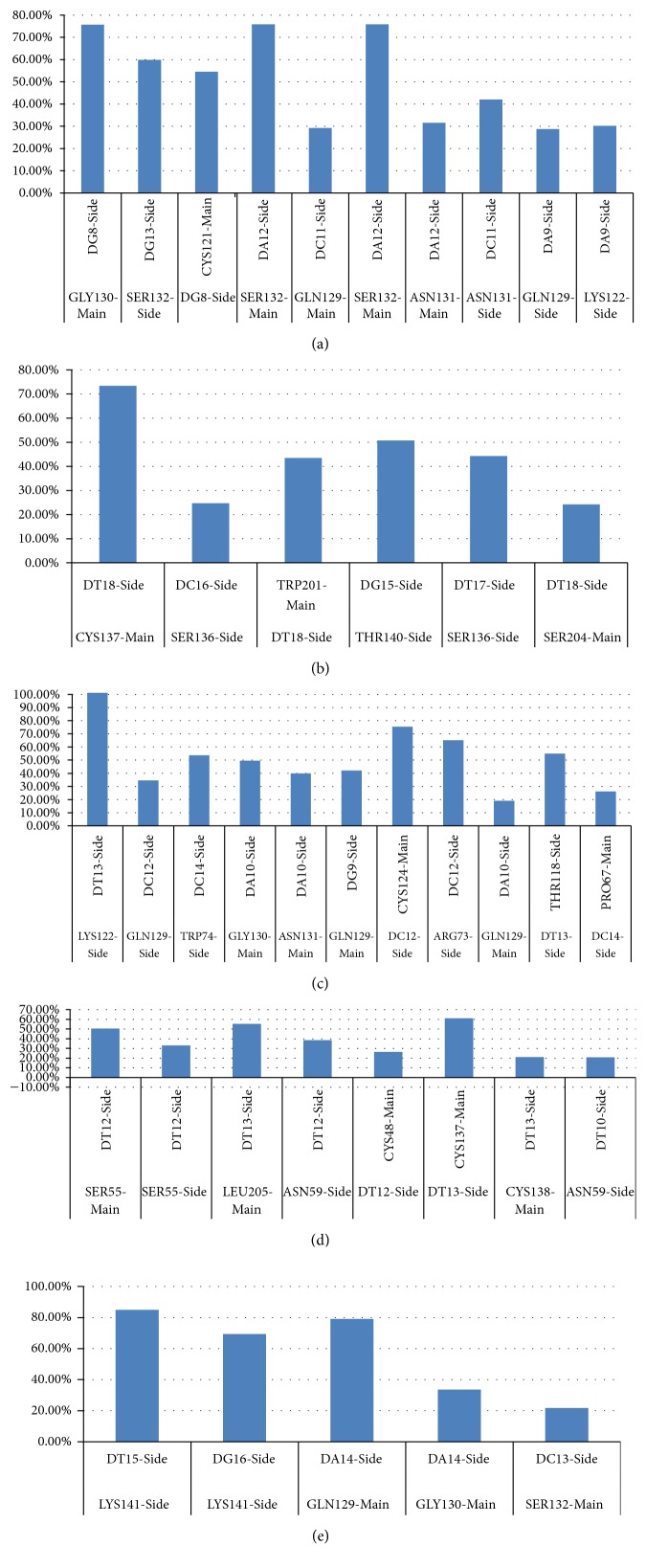
Histogram of intramolecular hydrogen bonds with occupancy percentage higher than 20% for each complex: (a) H01a, (b) H01b, (c) H02a, (d) H02b, and (e) H03a. Electron acceptors are on the top of x-axis while electron donors are at the bottom. H02a shows the highest number of aptamer-HBsAg intramolecular polar interactions, and its DT13 and Lys122 hydrogen bonds are the most stable as they exist for the whole 20 ns (100%).

The “Hydrogen Bonds” plugin in VMD software was used to visualize the dynamic hydrogen bonds formation in between the complex.  Figure 7 shows which residues that keep on contributing to the hydrogen bond formation, either as donors or as acceptors. High occupancy percentage is determined as the occupancy value higher than 20% while the occupancy value lower than 20%, although also recorded by VMD, is omitted due to the inefficient intermolecular hydrogen bonds formed. The result shows that several aptamers share the active binding residues, for example, GLN129, GLY130, and SER132 which are the important active binding residues for H01a, H02a, and H03a. They are located adjacent to each other, suggesting that any single amino acid backbone reorganization during the aptamer binding will affect the adjacent amino acids to coordinate closer towards the aptamer. This phenomenon is also observed on aptamer H01b-HBsAg complex, where the active binding residues are SER136, CYS137, and THR140.

In addition, aptamer H02b active binding residues are SER55 and LEU205 which are both located outside the “a” determinant region. The observation suggests that since the aptamer is a large macromolecules (nucleic acids) rather than drug-like small ligands, binding of the aptamer has to be inclusive of other potential conformational binding sites located outside the antigen epitope.

Some of the active binding residues are also be able to form hydrogen bonds by becoming the electron donor through their main and also side chains. H02a has the most active binding residues that give more than 20% occupancy of dynamic intermolecular hydrogen bonds, namely, LYS122, GLN129, GLY130, and ASN131 of the “a” determinant region residues and also ARG73 and TRP74 (Figure 7(c)). Overall, the important active binding residues in the conformational structure are LYS122, GLN129, GLY130, ASN131, SER132, SER136, CYS137, and THR140 from the “a” determinant region and also the residues located near to the region which is SER55, ARG73, TRP74, and LEU205.

#### 3.3.3. Molecular Mechanics Poisson-Boltzmann Surface Area (MMPBSA)

MMPBSA method has been widely applied and is considered as a reliable free energy simulation method to understand the protein-ligand binding interactions [32]. Another molecular mechanics energies method is implying generalized Bonn (GB) surface area continuum solvation, thus known as MMGBSA method in estimating the free energy binding of ligands to biological macromolecules. The free energy of a state, as described by Kollman and coworkers [33], is as follows:

(1) $$\begin{array}{c} ∆\overset{-}{G}=∆{\overset{-}{E}}_{MM}+∆{\overset{-}{G}}_{PBSA}-T∆S_{MM} \end{array}$$

where $∆\overset{-}{G}$ is the calculated average free energy, $∆{\overset{-}{E}}_{MM}$ is the average molecular mechanics energy, $∆{\overset{-}{G}}_{PBSA}$ is the solvation free energy, and −T∆*S*_*MM*_ represents the solute configuration entropy which can be determined by the quasi-harmonic analysis of the trajectory or by normal-mode analysis. $∆{\overset{-}{G}}_{PBSA}$ can be calculated with the Poisson-Boltzmann equation numerical solution and the estimate of the nonpolar free energy with a simple surface area term. As the polar part of $∆{\overset{-}{G}}_{PBSA}$ solvation term is able to be solved by rigorous computational calculations, generalized Born is an approximate solution of Poisson-Boltzmann that is faster to compute, of which the equation gives the electrostatic potential around a solute in a solution.

The MMGBSA method is considered computationally less intensive on the technical consideration, in comparison to MMPBSA. However, solution of the Poisson-Boltzmann equation has been treated as the gold standard and has been the basis for the development of GB parameters. Therefore MMPBSA method is considered superior in terms of accuracy although it is expected that these two methods are highly to yield comparable results when GB is parameterized properly. Nevertheless, there are no direct comparisons between these two important methods on large data sets [34]. PB employs a more rigorous algorithm than GB [35] but the GB parameters have always been optimized by fitting experimental data. It is difficult to mention which method is better as they show conflicting performance for different systems. Study by Lei Xu & Huiyong Sun et al., 2013 [36], shows that, for most cases, MMPBSA with the PBSA program and Lu's radii set gives better ranking results than MMGBSA with GB. Therefore, based on the positive results showed by other studies, MMPBSA method was selected to estimate the free-binding energy of the last 5 ns of the aptamer-HBsAg complexes.

**Figure 8:**
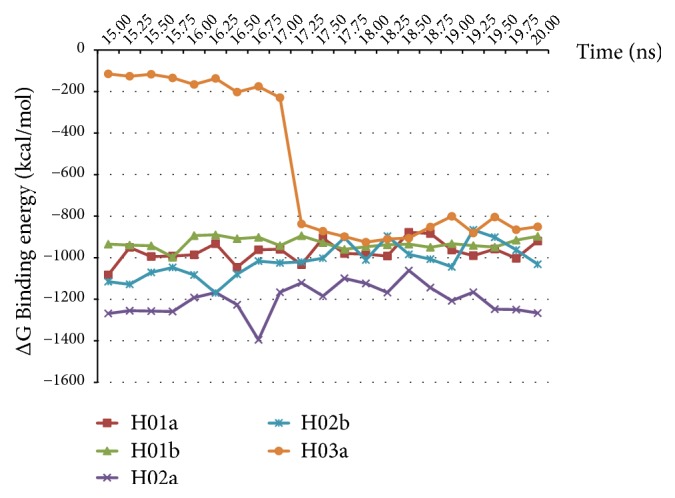
Total energy binding value for the last 5 ns of simulation (15-20 ns) as per calculated using the MMPBSA method. H02a shows the lowest total energy binding value which translated to the highest affinity towards HBsAg, while H03a gives highest total binding energy and less binding affinity towards HBsAg compared to other aptamers.

In current study, MMPBSA free energy simulation obtained for each aptamer and HBsAg complex for the last 5 ns of the simulation (15-20 ns duration) shows that while H01a, H01b, and H02b aptamer-HBsAg complex gives a stable energy value, H03a had been fluctuated (Figure 8). The total energy value of the complex only starts to decrease at 17 ns. This suggests that, for H03a, the aptamer binding is not fully stable for the whole course of the simulation. The finding is also in line with earlier study which suggested that aptamer H03 has a lower binding affinity compared to H01 and H02 and also with the binding energy value obtained by Vina. In contrast, aptamer H02a gives the lowest binding energy averaging at -1303.080 kJ/mol while maintaining a constant low binding energy for the last 5 ns of the simulation. Here, MMPBSA method is beneficial in determining the optimum aptamers sequence for the binding against HBsAg especially when all the aptamers gives a relatively low Vina docking score and have a similar GROMACS total energy (Table 4).

## 4. Conclusions

The thorough approach in determining the suitable aptamers against HBsAg by combining a multiple assessing strategies will tremendously improve the effort of aptamer screening. Our study shows that, by designing the three-dimensional structure of proteins and aptamers* de novo*, process of aptamers sequences selection against HBsAg can begin with the computational chemistry approach on a reasonable computational cost before expanding the study in the lab. Molecular docking are important to understand the aptamer-HBsAg complex binding orientation with the lowest ∆G binding energy, while molecular dynamics simulation shows the stability of the polar contacts between the complex. The active binding amino acids residues are the key players for the aptamer to bind on the surface of HBsAg. The intermolecular hydrogen bonds occupancy can be utilized to monitor the affinity of each screened aptamer against HBsAg, while MMPBSA approach is a highly convenient method to monitor the tightness of aptamer-protein binding complex especially during the end course of the MD simulation. This findings can be used to optimize the aptamers sequences obtained computationally into the* in vitro* method, in order to enhance the manipulation of the sequences. In addition, the experimental study to determine the binding affinity K_d_ of the complex is still required to support the computationally derived hypothesis. The correlation between the theoretical binding energy and experimental K_d_ is crucial to determine the most promising candidate for the high affinity HBsAg-aptamer synthesis.

## Figures and Tables

**Table 1:** Short single-strand DNA (ssDNA) aptamers sequence obtained based on the loop and hairpin structure available from each H01, H02, and H03.

No.	Aptamer name	Aptamer original	Sequence (5′-3′)*∗*
1	H01a	H01	-CCACAG**CGAACAGCG**CGGCAGG-
2	H01b	H01	-GCGGGACATAAT**AGTGCTTACT**ACGACCTGC-
3	H02a	H02	-GGGAATT**CGAGCTCG**GTACCC-
4	H02b	H02	-ATGAAG**TATTATTA**CCCAAT-
5	H03a	H03	-GCTCGCTG**CAGGCATG**CAAGC-

*∗*The bold and underlined alphabets were the sequences selected to design the three-dimensional conformation based on the hairpin formed in the two-dimensional structure.

**Table 2:** Transmembrane domain amino acid sequence from UniProt KB and predicted by I-TASSER.

Name	Transmembrane amino acid sequence			
Domain	(I)	(II)	(III)	(IV)

UniProt KB		80-98	170-199	205-225

I-TASSER	9-39	72-100	155-184	188-223

**Table 3:** Number of atoms in each aptamer, Vina docking score, and Root Mean Square (RMS) value of each docked aptamer before and after docking on HBsAg. The RMS value of each aptamer was determined by aligning before and after docking structure using PyMOL.

No.	Aptamer name	No. of atoms	RMS of initial and after docking structures	Vina score (kJ/mol)
1	H01a	222	6.978	-27.196
2	H01b	254	8.944	-31.798
3	H02a	254	8.006	-24.686
4	H02b	256	6.549	-24.686
5	H03a	255	6.133	-22.175

**Table 4:** Values of Vina score, calculated GROMACS total-energy, and calculated binding free energies using MMPBSA (kJ/mol) of loop and hairpin region truncated from each aptamer. H02a gives the lowest MMPBSA ∆E binding energy compared to other complexes.

Aptamer	Binding energy		
	Vina score (kJ/mol)	GROMACS Total energy (kJ/mol)	MM/PBSA ∆E binding (kJ/mol)
H01a	-27.196	-6.32094 x 10^5^	-971.119
H01b	-31.798	-7.73169 x 10^5^	-977.181
H02a	-24.686	-7.73238 x 10^5^	-1303.080
H02b	-24.686	-7.93305 x 10^5^	-974.453
H03a	-22.175	-7.71841 x 10^5^	-762.144

## Data Availability

The data in the study is available upon request.

## Supplementary Materials

Supplementary Materials Experimental protocols and result arrangements for the reported study. The protocol starts with each aptamer and HBsAg design and validations, preceded with molecular docking of aptamer against HBsAg using ADT Vina. The docked structures then undergo MD simulation for 20 ns using GROMACS. For the analysis, the RMS of aptamer before and after docking results from MD simulation; MMPBSA binding energy; active binding residues for 20 ns simulation and summary of each docking; MD total energy and MMPBSA binding energy were all presented in the study.

## References

[1] GBD 2013 Mortality and Causes of Death Collaborators (2015). Global, regional and national age-sex specific all-cause and cause-specific mortality for 240 causes of death, 1990-2013: a systematic analysis for the Global Burden of Disease Study 2013. *The Lancet*.

[2] Liang T. J. (2009). Hepatitis B: the virus and disease. *Hepatology*.

[3] Brunetto M. R. (2010). A new role for an old marker, HBsAg. *Journal of Hepatology*.

[4] Milich D. R., Leroux Roels G. G., Chisari F. V. (1983). Genetic regulation of the immune response to hepatitis B surface antigen (HBsAg). II. Qualitative characteristics of the humoral immune response to the a, d, and y determinants of HBsAg. *The Journal of Immunology*.

[5] Jolivet-Reynaud C., Lésenéchal M., O'Donnell B. (2001). Localization of hepatitis B surface antigen epitopes present on variants and specifically recognised by anti-hepatitis B surface antigen monoclonal antibodies. *Journal of Medical Virology*.

[6] Luo X., Mckeague M., Pitre S. (2010). Computational approaches toward the design of pools for the in vitro selection of complex aptamers. *RNA*.

[7] Chushak Y., Stone M. O. (2009). In silico selection of RNA aptamers. *Nucleic Acids Research*.

[8] Xi Z., Huang R., Li Z. (2015). Selection of HBsAg-specific DNA aptamers based on carboxylated magnetic nanoparticles and their application in the rapid and simple detection of hepatitis b virus infection. *ACS Applied Materials & Interfaces*.

[9] Xiao Z., Farokhzad O. C. (2012). Aptamer-functionalized nanoparticles for medical applications: Challenges and opportunities. *ACS Nano*.

[10] Yang J., Yan R., Roy A., Xu D., Poisson J., Zhang Y. (2015). The I-TASSER suite: protein structure and function prediction. *Nature Methods*.

[11] Arnold K., Bordoli L., Kopp J., Schwede T. (2006). The SWISS-MODEL workspace: a web-based environment for protein structure homology modelling. *Bioinformatics*.

[12] Laskowski R. A., MacArthur M. W., Moss D. S., Thornton J. M. (1993). PROCHECK: a program to check the stereochemical quality of protein structures. *Journal of Applied Crystallography*.

[13] Wiederstein M., Sippl M. J. (2007). ProSA-web: interactive web service for the recognition of errors in three-dimensional structures of proteins. *Nucleic Acids Research*.

[14] Mark P., Nilsson L. (2001). Structure and dynamics of the TIP3P, SPC, and SPC/E water models at 298 K. *The Journal of Physical Chemistry A*.

[15] Vanommeslaeghe K., Hatcher E., Acharya C. (2010). CHARMM general force field: a force field for drug-like molecules compatible with the CHARMM all-atom additive biological force fields. *Journal of Computational Chemistry*.

[16] Von Grotthuss M., Pas J., Wyrwicz L., Ginalski K., Rychlewski L. (2003). Application of 3D-Jury, GRDB, and verify3D in fold recognition. *Proteins: Structure, Function, and Genetics*.

[17] Fischer D. (2006). Servers for protein structure prediction. *Current Opinion in Structural Biology*.

[18] Zuker M. (2003). Mfold web server for nucleic acid folding and hybridization prediction. *Nucleic Acids Research*.

[19] Biesiada M., Purzycka K. J., Szachniuk M., Blazewicz J., Adamiak R. W. (2016). Automated RNA 3D structure prediction with RNAComposer. *RNA Structure Determination*.

[20] Forli S., Huey R., Pique M. E., Sanner M. F., Goodsell D. S., Olson A. J. (2016). Computational protein-ligand docking and virtual drug screening with the AutoDock suite. *Nature Protocols*.

[21] Trott O., Olson A. J. (2010). Software news and update AutoDock Vina: improving the speed and accuracy of docking with a new scoring function, efficient optimization, and multithreading. *Journal of Computational Chemistry*.

[22] Hess B., Kutzner C., van der Spoel D., Lindahl E. (2008). GROMACS 4: algorithms for highly efficient, load-balanced, and scalable molecular simulation. *Journal of Chemical Theory and Computation*.

[23] Hart K., Foloppe N., Baker C. M., Denning E. J., Nilsson L., MacKerell A. D. (2012). Optimization of the CHARMM additive force field for DNA: Improved treatment of the BI/BII conformational equilibrium. *Journal of Chemical Theory and Computation*.

[24] Kumari R., Kumar R., Open Source Drug Discovery Consortium, Lynn A. (2014). g_mmpbsa: a GROMACS tool for high-throughput MM-PBSA calculations. *Journal of Chemical Information and Modeling*.

[25] Gazina E., Gallina A., Milanesi G. (1996). Common localization of retention determinants in hepatitis B virus L protein from different strains. *Journal of General Virology*.

[26] Glebe D., Bremer C. M. (2013). The molecular virology of hepatitis B virus. *Seminars in Liver Disease*.

[27] Niazi S., Purohit M., Sonawani A., Niazi J. H. (2018). Revealing the molecular interactions of aptamers that specifically bind to the extracellular domain of HER2 cancer biomarker protein: An in silico assessment. *Journal of Molecular Graphics and Modelling*.

[28] Cao J., Pham D. K., Tonge L., Nicolau D. V. (2002). Predicting surface properties of proteins on the Connolly molecular surface. *Smart Materials and Structures*.

[29] Jeddi I., Saiz L. (2017). Three-dimensional modeling of single stranded DNA hairpins for aptamer-based biosensors. *Scientific Reports*.

[30] Roy S., Thakur A. R. (2010). 20ns molecular dynamics simulation of the antennapedia homeodomain-dna complex: water interaction and dna structure analysis. *Journal of Biomolecular Structure and Dynamics*.

[31] Nguyen B., Wilson W. D. (2009). The effects of hairpin loops on ligand-DNA interactions. *The Journal of Physical Chemistry B*.

[32] Wang C., Greene D. A., Xiao L., Qi R., Luo R. (2018). Recent developments and applications of the MMPBSA method. *Frontiers in Molecular Biosciences*.

[33] Kollman P. A., Massova I., Reyes C. (2000). Calculating structures and free energies of complex molecules: combining molecular mechanics and continuum models. *Accounts of Chemical Research*.

[34] Lee M. C., Yang R., Duan Y. (2005). Comparison between Generalized-Born and Poisson-Boltzmann methods in physics-based scoring functions for protein structure prediction. *Journal of Molecular Modeling*.

[35] Sitkoff D., Sharp K. A., Honig B. (1994). Accurate calculation of hydration free energies using macroscopic solvent models. *The Journal of Physical Chemistry C*.

[36] Xu L., Sun H., Li Y., Wang J., Hou T. (2013). Assessing the performance of MM/PBSA and MM/GBSA methods. 3. the impact of force fields and ligand charge models. *The Journal of Physical Chemistry B*.

