# Supplementary material for: In Silico Screening of Aptamers Configuration against Hepatitis B Surface Antigen
Source: Adv Bioinformatics. 2019 Jun 26;2019:6912914. doi: 10.1155/2019/6912914 (PMC6617924; doi:10.1155/2019/6912914)
Supplement: Supplementary Materials — Experimental protocols and result arrangements for the reported study. The protocol starts with each aptamer and HBsAg design and validations, preceded with molecular docking of aptamer against HBsAg using ADT Vina. The docked structures then undergo MD simulation for 20 ns using GROMACS. For the analysis, the RMS of aptamer before and after docking results from MD simulation; MMPBSA binding energy; active binding residues for 20 ns simulation and summary of each docking; MD total energy and MMPBSA binding energy were all presented in the study. [file 6912914.f1.pdf]

Supplementary Figure

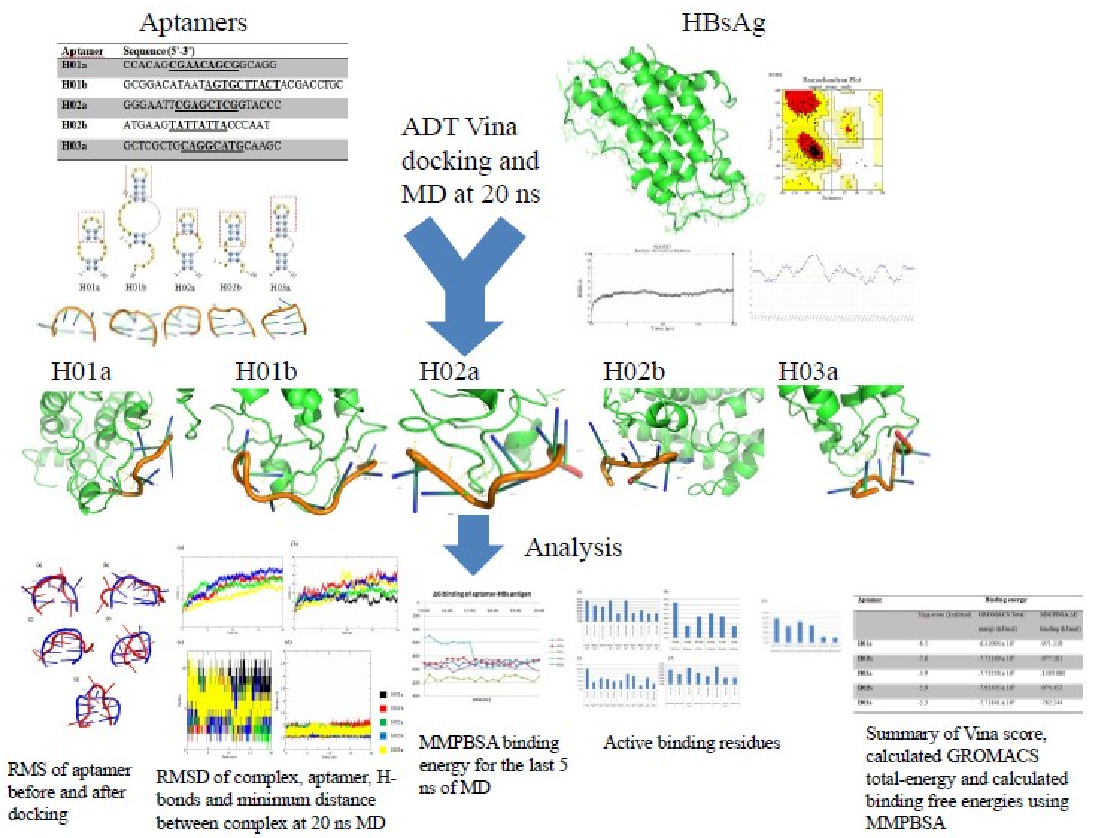

Experimental protocols and result arrangements for the reported study. The protocol starts with each aptamer and HBsAg design and validations, preceded with molecular docking of aptamer against HBsAg using ADT Vina. The docked structures then undergo MD simulation for 20 ns using GROMACS. For the analysis, the RMS of aptamer before and after docking, results from MD simulation; MMPBSA binding energy; active binding residues for 20 ns simulation and summary of each docking; MD total-energy and MMPBSA binding energy were all presented in the study.
